# Efficacy of inactivated SARS-CoV-2 vaccination in pediatric hematology/oncology patients: a real-world study

**DOI:** 10.1007/s12519-023-00737-3

**Published:** 2023-07-31

**Authors:** Jing Miao, Jing-Ying Zhang, Juan Liang, Fen-Ying Zhao, Hua Song, Wei-Qun Xu, Yong-Min Tang, Xiao-Jun Xu, Qiang Shu

**Affiliations:** 1 Children’s Hospital, Zhejiang University School of Medicine, Hangzhou 310052, China; 2 National Clinical Research Center for Child Health, Hangzhou, China

Severe acute respiratory syndrome coronavirus 2 (SARS-CoV-2) has been spreading globally since its initial outbreak in 2019. Substantial evidence has revealed that children who are receiving antitumor therapy or hematopoietic stem cell transplantation (HSCT) present higher rates of severe illness and mortality [1]. It is important to identify infected children with hematology and oncology diseases who have the tendency to develop worse outcomes of coronal virus disease 2019 (COVID-19) during the early stage of COVID-19. Recent studies have demonstrated that vaccination against SARS-CoV-2 in adults can reduce the likelihood of infection and reduce the severity and mortality of COVID-19 when SARS-CoV-2 breakthrough infection occurs [2–4]. However, the effectiveness of SARS-CoV-2 vaccination in children with hematology and oncology diseases is poorly defined due to the lack of relevant data, which might lead to vaccine hesitancy [5, 6].

This retrospective study enrolled a total of 190 pediatric hematology and oncology patients with clear vaccination status who were infected with SARS-CoV-2 between December 1, 2022, and January 19, 2023. All of these children had received specialty treatment in the Department of Hematology & Oncology, Children's Hospital of Zhejiang University School of Medicine. Detailed baseline and outcome data were obtained through clinical medical records, attending physicians, and guardians. Laboratory data within three days after SARS-CoV-2 infection and at the peak of the disease were collected. The patient was diagnosed with COVID-19 by viral nucleic acid or antigen testing. COVID-19 was classified into four categories based on disease severity according to guidelines published by the National Health Commission of China on March 14, 2022: (1) mild: symptomatic infection of the upper respiratory tract (URT) without any pneumonia manifestations in radiology; (2) moderate: radiological proof of pulmonary involvement but oxygen saturation > 93% without any oxygen support; (3) severe: any one of the following signs: shortness of breath, oxygen saturation ≤ 93%, dyspnea, convulsion or disturbance of consciousness, food refusal or feeding difficulties accompanied by signs of dehydration, high-resolution computed tomography (HRCT) of chest showing bilateral or multiple lobular infiltration, > 50% lung imaging progress in a short period, or pleural effusion; and (4) critical: any one of the following signs: shock, respiratory failure requiring mechanical ventilation, or organ failure requiring intensive care unit. This study protocol was reviewed and approved by the Ethics Committee of Children's Hospital, Zhejiang University School of Medicine (2023-IRB-0032-P-01) as a retrospective study. Informed consent was obtained from the guardians.

The baseline characteristics are summarized and the comparison is presented in Table 1. The median age of the population included in this study was 6.7 years, including 65 females (34.2%). Among them, 140 children (73.7%) were diagnosed with acute leukemia, and 24 (12.6%) had received HSCT. One hundred thirteen children (59.5%) were receiving ongoing active chemotherapy. The median value of the presumed incubation period (the time from the first exposure to a symptomatic patient to the first onset of symptoms) was three days. There were 144 children (75.8%) who developed mild illness, 40 (21.1%) with moderate illness, and 6 (3.2%) with severe or critical diseases, among whom one child died of acute respiratory distress syndrome.

**Table 1:** Clinical characteristic of children

Variables	Total (*n* = 190)	Vaccinated group (*n* = 83)	Unvaccinated group (*n* = 107)	*P* value
Age, median (IQR)	6.7 (5.2–10.3)	7.0 (5.5–10.9)	6.5 (4.9–9.9)	0.146
Sex				0.538
Female	65 (34.2%)	26 (31.3%)	39 (36.4%)	
Male	125 (65.8%)	57 (68.7%)	68 (63.6%)	
Height (cm)	119.5 (109.0–140.5)	120.0 (112.5–144.0)	119.0 (105.8–140.0)	0.073
Weight (kg)	22.7 (18.5–32.0)	23.0 (20.0–33.0)	22.0 (18.0–32.0)	0.416
BMI (kg/m^2^)	16.3 (14.8–18.0)	16.2 (14.4–17.7)	16.4 (15.1–18.1)	0.141
Diagnosis, *n* (%)				0.120
ALL	140 (73.7%)	66 (79.5%)	74 (69.1%)	
AML	24 (12.6%)	8 (9.6%)	16 (15.0%)	
Lymphoma	9 (4.7%)	6 (7.2%)	3 (2.8%)	
Others	17 (8.9%)	3 (3.6%)	14 (13.1%)	
Active chemotherapy	113 (59.5%)	51 (61.4%)	62 (58.0%)	0.657
Severity				0.003
Mild	144 (75.8%)	73 (88.0%)	71 (66.4%)	
Moderate	40 (21.1%)	7 (8.4%)	33 (30.8%)	
Severe	4 (2.1%)	2 (2.4%)	2 (1.9%)	
Critical	2 (1.1%)	1 (1.2%)	1 (0.9%)	
Presumed incubation period	3 (2–5)	4 (3–5)	3 (2–5)	0.284
WBC	2.28 (1.45–3.20)	2.31 (1.21–4.15)	2.20 (1.58–3.55)	0.852
Lymphocyte (× 10^9^/L)	0.64 (0.30–1.15)	0.56 (0.30–1.00)	0.73 (0.32–1.47)	0.147
Neutrophil (× 10^9^/L)	1.11 (0.60–2.18)	1.26 (0.42–2.43)	1.01 (0.61–2.17)	0.622
PLT (× 10^9^/L)	145 (81–233)	140 (90–219)	159 (79–235)	0.944
Hb (g/L)	111 (98–122)	111 (98–122)	110 (95–123)	0.795
CRP (mg/L)	1.58 (0.50–5.76)	1.85 (0.5–6.3)	1.41 (0.50–5.04)	0.931

*IQR* interquartile ranges, *BMI* body mass index, *ALL* acute lymphoblastic leukemia, *AML* acute myeloid leukemia, *WBC* white blood cell, *PLT* platelet, *Hb* hemoglobin, *CRP* C-reactive protein

The *P* value shows the comparison result between vaccinated group and unvaccinated group

All 190 children were categorized into vaccinated and unvaccinated groups. A total of 107 children had never been vaccinated against SARS-CoV-2, while 83 children had received at least one dose of vaccine, all of whom were immunized with inactivated vaccines. All the patients in the vaccinated group had received SARS-CoV-2 vaccination before they developed hematology and oncology diseases. There were no significant differences in age, sex, height, weight, body mass index, hematologic malignancy types, or active chemotherapy status between the two groups (for all, *P* > 0.05). However, there was a higher rate of non-mild cases in the unvaccinated group than in the vaccinated group [risk ratio (RR): 2.79, 95% confidence interval (CI) 1.47–5.29, *P* = 0.001], while there was no significant difference in the hospitalization rate (*P* = 0.678) (Fig. 1a). Furthermore, Spearman analysis was performed, and negative correlations were observed between the number of vaccinations and the severity of COVID-19 (correlation coefficient: − 0.244, *P* = 0.001).

**Fig. 1:**
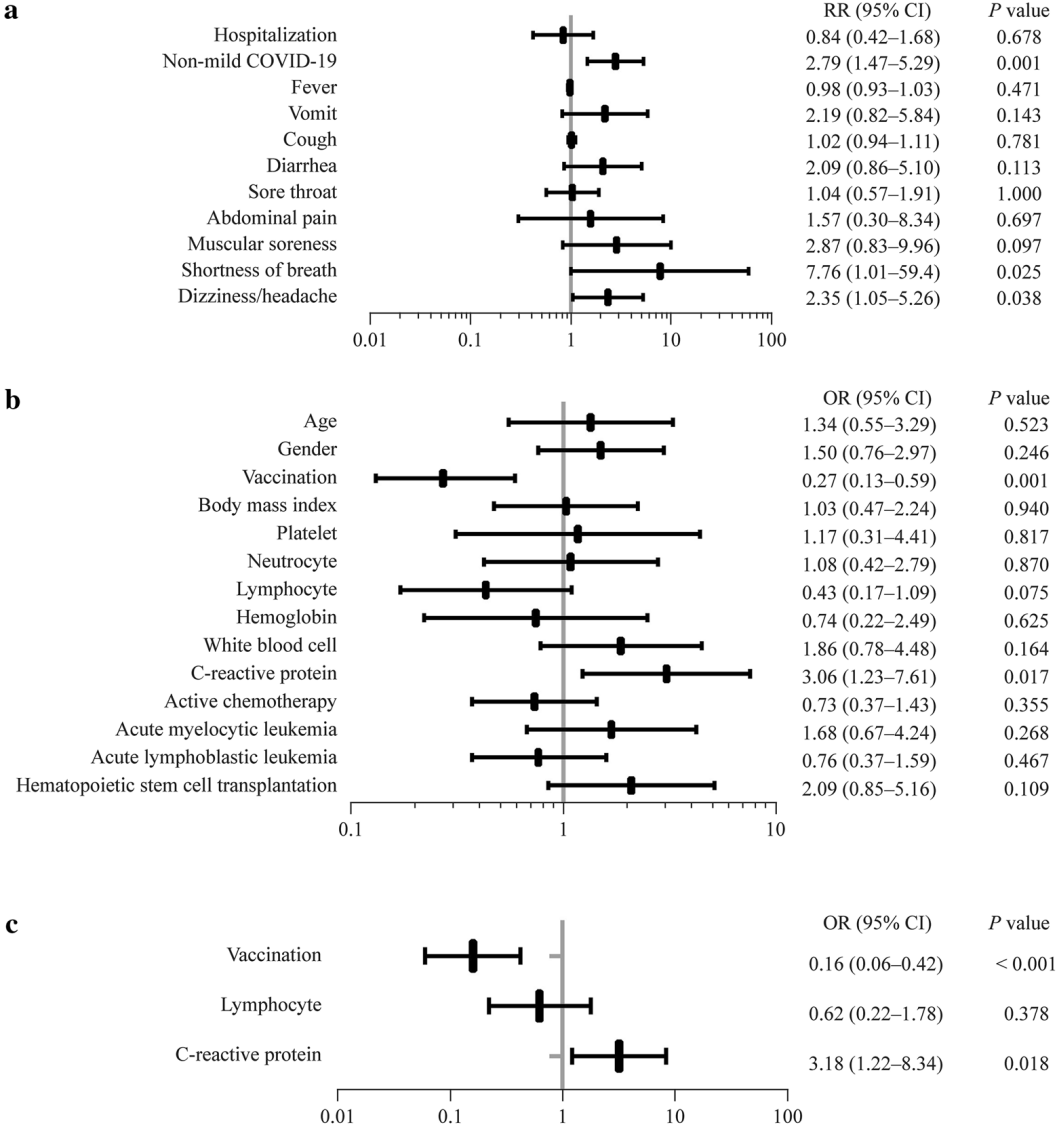
Relative risk (RR) of COVID-19-related symptoms  and outcomes and risk factors for non-mild COVID-19. **a** The RR of COVID-19-related symptoms and outcomes in the vaccinated and unvaccinated groups; **b** The results of univariable logistic regression analysis between the mild COVID-19 group and the non-mild COVID-19 group. The continuous variables were recategorized as dichotomous variables: age, ≤ 12 years vs. < 12 years; body mass index, ≤ 15  vs.  15; platelet, < 300 × 10^9^/L vs. ≥ 300 × 10^9^/L; neutrophil, < 0.6 × 10^9^/L vs. ≥ 0.6 × 10^9^/L; lymphocyte, < 2 × 10^9^/L vs. ≥ 2 × 10^9^/L; hemoglobin, < 130 g/L vs. ≥ 130 g/L; white blood cell, < 4 × 10^9^/L vs. ≥ 4 × 10^9^/L; C-reactive protein, < 1 mg/L vs. ≥ 1 mg/L. **c** The results of multivariable logistic regression analysis between the mild COVID-19 group and the non-mild COVID-19 group. The continuous variables were recategorized as dichotomous variables: lymphocytes, < 2 × 10^9^/L vs. ≥ 2 × 10^9^/L; C-reactive protein, < 1 mg/L vs. ≥ 1 mg/L. *COVID-19* coronavirus disease 2019, *RR* risk ratio, *OR* odds ratio

In terms of COVID-19-related symptoms, the incidence of shortness of breath and headache/dizziness was higher in the unvaccinated group than in the vaccinated group (shortness of breath RR: 7.76, 95% CI 1.01–59.4, *P* = 0.025; headache/dizziness RR: 2.35, 95% CI 1.05–5.26, *P* = 0.038), and there were no significant differences in the incidence of fever, cough, vomiting, abdominal pain, diarrhea, muscle soreness, and sore throat (for all, *P* > 0.05). (Fig. 1a).

Laboratory data at the peak of the disease and presumed incubation period were also analyzed, and the results showed that there were no significant differences in the presumed incubation period and levels of white blood cell, lymphocyte, neutrophil, platelet, C-reactive protein (CRP) and hemoglobin (*P* > 0.05) (Table 1).

Meanwhile, 190 children were categorized into the mild COVID-19 group (*n* = 144) and the non-mild COVID-19 group (*n* = 46) according to the severity of COVID-19. Univariable and multivariable logistic regression analyses were used to analyze clinical characteristics and laboratory data (within three days after SARS-CoV-2 infection). Through univariable logistic regression, we identified two factors (vaccination and CRP) with *P* < 0.05 and one factor (lymphocyte) with *P* < 0.1 (Fig. 1b). As previous studies revealed that lymphocytes were independent risk factors [1, 7], we included these three factors in multivariable logistic regression analysis, which revealed that vaccination [odds ratio (OR): 0.16, 95% CI 0.06–0.42, *P* < 0.001] was a protective variable, and elevated CRP (OR 3.18, 95% CI 1.22–8.34, *P* = 0.018) was a risk factor (Fig. 1c).

Previous studies showed that nearly 20% of parents were hesitated about vaccination, which was related to ignorance about vaccine efficacy [5, 8, 9]. Our findings filled a gap in the study of the effectiveness of vaccines and might enhance the confidence in vaccination in children with hematology and oncology diseases. Vaccination against SARS-CoV-2 can significantly reduce the incidence of shortness of breath and headache or dizziness and significantly reduce the severity of COVID-19 in children with hematologic diseases, which is similar to the data in adult cancer patients [2, 10, 11]. Moreover, a trend of severity reduction was observed as the number of vaccinations increased. These findings suggest that we can reduce the severity and mortality of COVID-19 in children with hematologic diseases through booster vaccination during the Omicron pandemic [12].

In this study, vaccination did not reduce hospitalization rates, which is different from other studies [13]. This might be related to the fact that most of the children included in this study were undergoing active chemotherapy, and chemotherapy-related hospitalizations interfered with the results. In addition, vaccination seemed not to affect the incubation period of COVID-19 in children with hematologic diseases.

There are some limitations in this study. It is a single-center retrospective study and is prone to recall bias and admission bias. This study only assessed limited laboratory data (the levels of blood cells and CRP), possibly overlooking the impact of other factors. Finally, the protective effects of different vaccination brands were not compared.

In conclusion, vaccination against SARS-CoV-2 can significantly improve clinical outcomes related to COVID-19 in children with hematology/oncology diseases. SARS-CoV-2 vaccination and booster doses were recommended for children with hematologic diseases. Children with elevated CRP at the early stage of COVID-19 are significantly more likely to develop non-mild COVID-19 as the disease progresses.

## Data Availability

The datasets generated during and/or analyzed during the current study are available from the corresponding author on reasonable request.
